# Click-Triggered Bioorthogonal Bond-Cleavage Reactions

**DOI:** 10.1007/s41061-025-00492-1

**Published:** 2025-06-14

**Authors:** Patrick Keppel, Sebastian Hecko, Hannes Mikula

**Affiliations:** Institute of Applied Synthetic Chemistry, TU Wien, Getreidemarkt 9, 1060 Vienna, Austria

**Keywords:** Bioorthogonal chemistry, Click chemistry, Click-to-release, Cycloaddition, Elimination

## Abstract

Bioorthogonal bond-cleavage reactions have evolved into powerful tools for chemical biology, representing a promising strategy for achieving controlled release of molecules under physiologically relevant conditions, even in living organisms. Since their discovery, significant efforts have been invested in the development and understanding of the underlying chemistries to enhance the click-to-release performance, biocompatibility, and stability of bioorthogonal tools. In this review, we aim to provide a concise overview of click-triggered bioorthogonal bond-cleavage reactions, with an emphasis on the mechanisms and characteristics of the most commonly applied click-to-release chemistries.

## Introduction

First coined in 2003 by Bertozzi [1], bioorthogonal chemistry refers to controlled and selective chemical reactions that neither interact nor interfere with biological systems. Reaction partners must be inert to the variety of predominant functional groups found in biological systems, stable under physiological conditions, and especially for in vivo use, nontoxic to cells and organisms. Furthermore, reaction kinetics play an indispensable role in tackling biologically relevant concentrations in the submicromolar range [2]. These conditions put high demands on the chemistry itself and therefore limit the scope of available reactions.

Since their introduction, bioorthogonal chemistries mainly focused on ligation reactions, such as for labeling [3], tracking [4], and imaging applications [5]. Moving toward controlled bioorthogonal bond-cleavage, the field expanded massively with the development of redox-based [6–10], transition metal-mediated [11], and click-triggered reactions, leading to the release of an attached payload. In this review, we summarize the developments of click-triggered bioorthogonal bond-cleavage chemistries with a particular focus on reactions initiated by cycloadditions. We discuss the underlying mechanisms, with an emphasis on initial click and subsequent release kinetics.

**Fig. 1:**
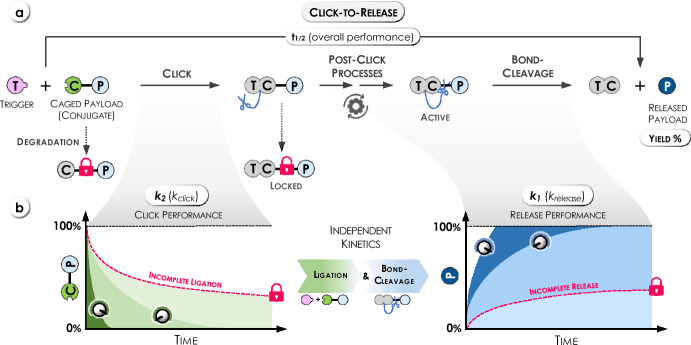
**a** Concept of click-triggered bioorthogonal bond-cleavage reactions (click-to-release) and relevant parameters (rate constants *k*_*2*_ and *k*_*1*_, and release yield) of the individual steps. Degradation of the reagents or formation of non-releasing byproducts limit the overall efficiency. **b** Illustrations of click and release proceeding (independently) at different rates

In all examples, the first step involves the click reaction (ligation) of a caging group-payload conjugate (C-P) with a trigger (T). In some cases, post-click processes (e.g., tautomerization) are required to obtain a TC-P-conjugate, which can undergo elimination or cyclization, facilitating the release of the attached payload (P). However, several undesired pathways can compete with the click-to-release process, including the formation of non-releasing TC-P byproducts or the degradation of the caged payload (C-P) (Fig. 1a). The overall chemical performance (t_1/2_, half-life of the click-to-release reaction) is thereby not only dependent on the reaction kinetics of the initial bimolecular ligation (*k*_*2*_), but also on all subsequent unimolecular processes (*k*_*1*_, Fig. 1b). Most crucially, fast ligation does not necessarily translate into a fast and efficient payload release. In some cases, these processes are inversely coupled, and modification of the chemical structures of the reactants is necessary to tune the individual kinetics and control of the overall process. In general, efficient click-to-release requires both fast ligation and fast bond-cleavage.

**Fig. 2:**
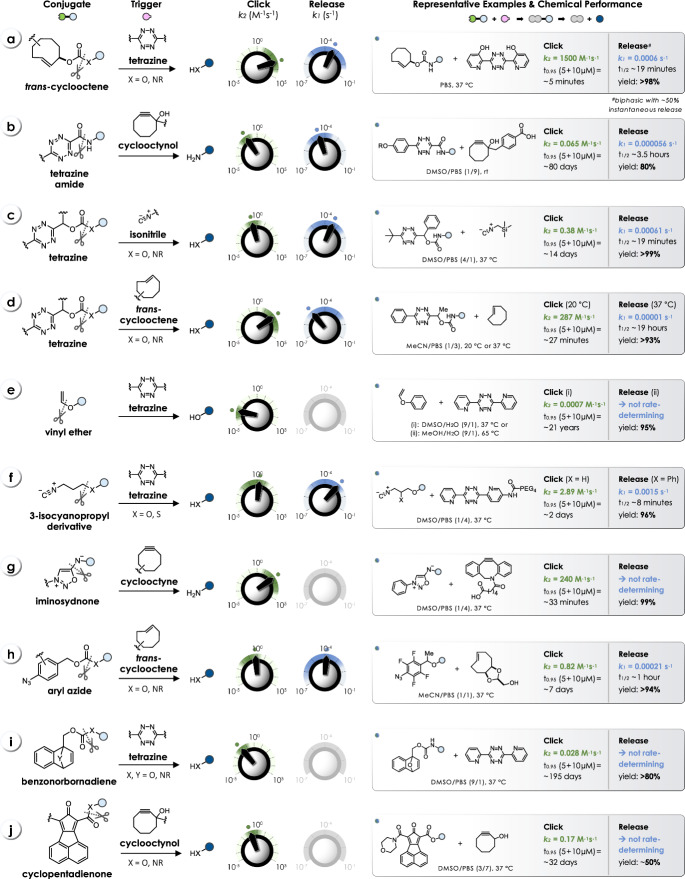
Overview of click-triggered bioorthogonal bond-cleavage reactions and their performance parameters, i.e., click kinetics (*k*_*2*_) and release kinetics (*k*_*1*_). For selected reactions, the rate constants (*k*_*2*_, *k*_*1*_) were used to calculate the time required to reach 95% click (t_0.95_) at reactant concentrations of 5 µM and 10 µM, as well as the half-life (t_1/2_) of the subsequent release reaction. A detailed list of *k*_*2*_ and *k*_*1*_ values is provided at the end of the individual chapters of this review

Various click-triggered bioorthogonal bond-cleavage reactions have been developed, employing different pairs of caging groups and triggers to achieve controlled release of the payload (Fig. 2). The chemistry of these reactions and the underlying mechanisms will be discussed in the respective chapters of this review.

The click-to-release kinetics of bioorthogonal reactions can span from several hours to days. To date, there are only a limited number of methods available that achieve both fast (within minutes rather than hours) and efficient (> 95%) click-triggered release of the payload at biologically relevant concentrations of both reactants. It is important to note that reaction kinetics are highly dependent on experimental conditions such as solvent, temperature, and pH, which complicates the direct comparison of kinetic values both within each set of reactants and across different click chemistries.

## Tetrazine-Triggered Bioorthogonal Cleavage of *trans*-Cyclooctene Conjugates

**Fig. 3:**
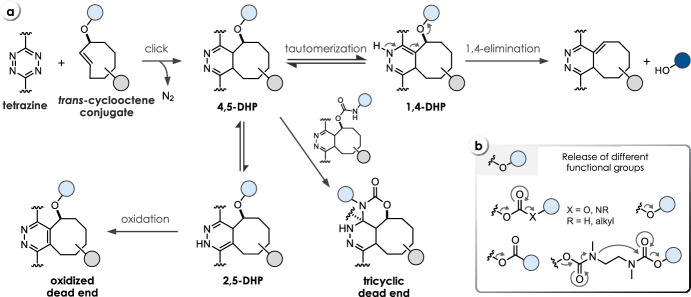
**a** Reaction mechanism of tetrazine-triggered cleavage of *trans*-cyclooctenes. After initial ligation, the 4,5-dihydropyridazine (4,5-DHP) tautomerizes either to a 1,4- or a 2,5-dihydropyridazine (1,4- or 2,5-DHP) tautomer, with only the 1,4-DHP leading to release of the payload via 1,4-elimination. Mechanistic studies revealed the formation of a tricyclic and oxidative dead-end species as the reason for poor release. **b** Release of different functional groups, including primary and secondary amines (from rTCO-carbamates), carboxylic acids (from rTCO-esters), alcohols (from rTCO-carbonates or rTCO-ethers), and phenols (from rTCO-carbamates modified with a self-immolative *N*,*N*’-dimethylethylenediamine linker)

Considered a breakthrough in the field, bioorthogonal click-to-release chemistry, first reported in 2013 by Robillard and co-workers [12], meets the criteria of bioorthogonality [13] and shows high reaction rates. A *trans*-cyclooctene functionalized at the allylic position (release-TCO, rTCO) reacts with a 1,2,4,5-tetrazine (Tz) in an initial inverse electron demand Diels–Alder (IEDDA) cycloaddition step followed by a sequence of electron cascade steps, leading to the release of a payload (Fig. 3a). Therein, the 4,5-dihydropyridazine (4,5-DHP) click product rapidly tautomerizes to 1,4- and 2,5-dihydropyridazines (1,4-DHP and 2,5-DHP), with the 1,4-DHP tautomer being able to release the attached payload via a 1,4-elimination. Due to steric reasons, the axial isomer of rTCO is > 100-fold more reactive compared with the equatorial isomer. Despite suitable biocompatibility using bis-alkyl or alkyl-aryl Tz as triggers, the overall chemical performance is limited due to reduced click reactivity as well as insufficient and/or slow release. Nevertheless, this reaction has successfully been applied in recent years for the in vivo activation of antibody–drug-conjugates [14, 15], prodrugs [16–22], and proteins [23]. In a detailed mechanistic investigation of the click-to-release mechanism (Fig. 3a), Carlson et al. discovered the formation of a tricyclic dead-end isomer as the reason for poor release when reacting bis-alkyl Tz with commonly used rTCO-carbamates (**C1**, Table 1) [24]. Applying *N*-methyl-substituted TCO-carbamates (**C2**) prevents the formation of this dead-end isomer, enabling complete release of secondary amines. More recently, this approach has been extended to release phenols, carboxylic acids, and to some extent alcohols (Fig. 3b) [17, 25–27]. While release of phenols and carboxylic acids could be facilitated using a self-immolative linker strategy [17] and rTCO-esters (**C3**) [26], respectively, the elimination of rTCO-ethers (**C4**) to release alcohols upon Tz ligation proceeds only very slowly [26]. The stability of otherwise labile rTCO-esters (e.g., 87–96% degradation after 5 h in serum [26]) can be improved significantly by increasing steric hindrance at the payload site (e.g., 5% free drug after 24 h in 10% plasma) [27]. rTCO-carbonates (**C5**) allow for sufficient release of alcohols but show only limited stability (e.g., 100% fragmentation after 5 h in serum) [26]. Nevertheless, this caging strategy was used to design click-activatable prodrugs of paclitaxel and etoposide [22].

**Fig. 4:**
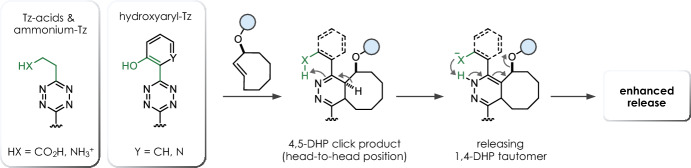
Enhanced cleavage of *trans*-cyclooctenes upon click with tetrazine-acids, ammonium-functionalized Tz, and hydroxyaryl tetrazines through intramolecular proton-exchange [24, 29–31]

The release step is a complex process, with many factors influencing the final outcome [24, 26, 28]. Most importantly, the structure of the Tz plays a crucial role in the reaction. Electron-withdrawing substituents on the Tz increase the reaction rate of the cycloaddition, while such substituents decelerate the formation of the releasing 1,4-DHP tautomer, leading to a slower release of the caged payload. However, unsymmetric tetrazines modified with an electron-withdrawing substituent and an alkyl group offer accelerated release abilities (> 80%, 30 min) [28]. In general, tautomerization can be promoted under acidic conditions [24], a finding that led to the development of Tz-acids that facilitate accelerated tautomerization through intramolecular participation, thereby achieving complete release under physiological conditions (Fig. 4). Alternatively, ammonium-functionalized tetrazines were shown to improve release kinetics but are limited in release efficiency (~ 80%) [29]. Hydrogen bonding between the NH_3_^+^ group and the TCO-carbamate favors the formation of the head-to-head click product, and subsequent intramolecular protonation promotes tautomerization (Fig. 4). Very recently, we introduced hydroxylated aryl tetrazines (including highly reactive bis-pyridyl-Tz scaffolds) that achieve complete cleavage within minutes by intramolecular proton-exchange, efficiently accelerating post-click tautomerization and elimination (Fig. 4) [30]. Vrabel and co-workers significantly accelerated the release step by developing sulfonated bis-hydroxyphenyl-tetrazines and further demonstrated the versatility of these bioorthogonal tools by derivatizing them into Tz-sulfonamides suitable for intracellular applications. [31]. Due to the inability to control click orientation, symmetric tetrazines modified with directing groups are generally used to prevent exclusive formation of a nonreleasing head-to-tail product and to facilitate tautomerization between the post-click DHP-isomers.

**Fig. 5:**
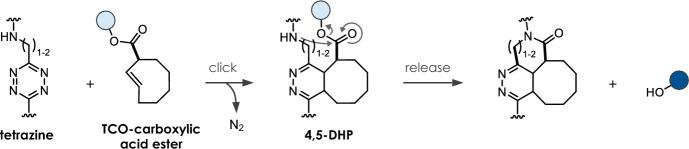
Tetrazine-triggered release of alcohols and phenols from TCO-carboxylic acid esters via intra-molecular lactamization

An alternative cleavage strategy was developed by Royzen and coworkers [32], who reported the release of benzylic alcohols and phenols from TCO-carboxylic acid esters (**C6**) upon ligation with symmetric Tz-amines and subsequent intramolecular lactamization (Fig. 5, Table 1). While reacting 50 µM TCO with 500 µM Tz achieved > 80% release after approximately 3 h, no specific reaction rates were reported [32].

The unique properties of the bioorthogonal pyridazine elimination were successfully extended to cleavable TCO linkers, with additional handles installed on the TCO scaffold. Using this approach, Robillard and co-workers developed a cleavable TCO linker (**C7**, cTCO, Table 1) [14] with an additional carboxylic acid functionality to enable further conjugation. cTCO provides increased release performance compared with rTCO [24] but lower release efficiency when using ammonium-modified Tz (50%) [17]. To reduce synthetic efforts, we developed the dioxolane-fused cleavable TCO linker **C8** (dcTCO), which offers high stability (99% TCO after 4 days in PBS at 37 °C) and high release performance (85%) [18]. The groups of Rutjes and Robillard developed synthetic strategies to access cleavable cyclopropane-fused TCO linkers (**C9**) [33, 34].

**Fig. 6:**
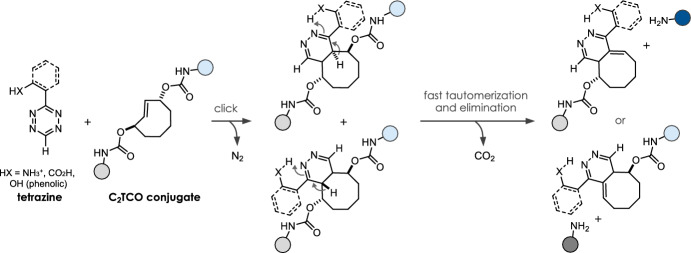
Omnidirectional bioorthogonal cleavage of C_2_TCO conjugates independent of Tz/TCO click orientation

To enhance release performance and circumvent the formation of non-releasing tautomers, we have developed the cleavable *C*_2_‑symmetric *trans*-cyclooctene **C10** (C_2_TCO, Fig. 6, Table 1), which shows excellent release performance and high stability (> 97% after 48 h in cell growth medium including 10% fetal bovine serum, 37 °C) [35]. On the basis of its C_2_ symmetry, reaction with Tz-acids, ammonium-functionalized Tz, and tetrazines equipped with a directing hydroxyaryl group leads to fast and complete bioorthogonal cleavage, irrespective of the Tz/TCO click orientation [30, 35].

**Table 1:**
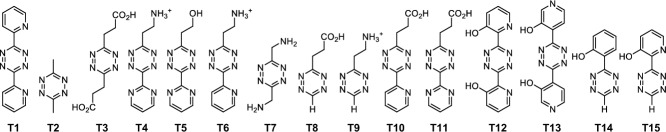

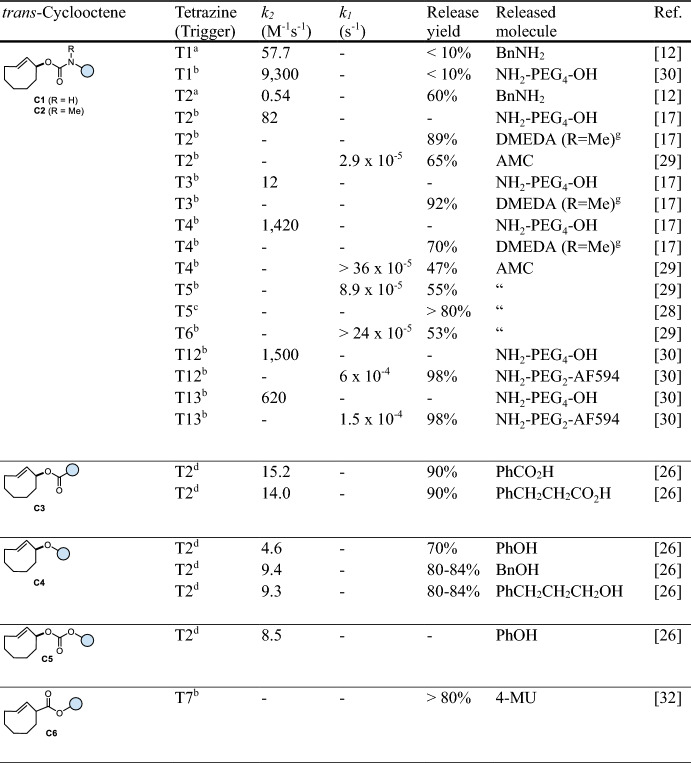

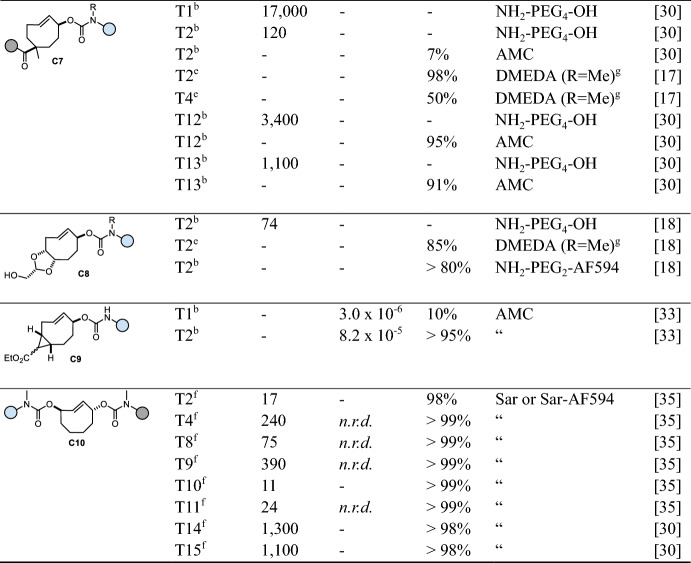
Chemical performance (*k*_*2*_, *k*_*1*_, release yield) of tetrazine-triggered click-to-release of *trans*-cyclooctenes

*AF594* Alexa Fluor 594; *AMC* 7-amino-4-methylcoumarin; *Bn* benzyl; *4-MU* 4-methylumbelliferone; *n.r.d.* not rate-determining; *Sar* sarcosine

Reaction conditions: ^a^ MeCN, 20 °C; ^b^ PBS, 37 °C; ^c^ H_2_O/DMSO (1/1), 37 °C; ^d^ PBS/MeCN (3/1), 20 °C; ^e^ PBS/DMSO (9/1), 37 °C; ^f^ phosphate buffer (pH 7.4), 37 °C. Unless otherwise noted, the DMSO content is <4%; ^g^ *N*,*N*´-dimethylethylenediamine (DMEDA) as self-immolative linker (for subsequent release of phenolic compounds)

## Click-Triggered Bioorthogonal Cleavage of Tetrazine Conjugates

### Cyclooctyne-Triggered Cleavage of Tetrazine Conjugates (*Click-Cyclize-Release*)

**Fig. 7:**
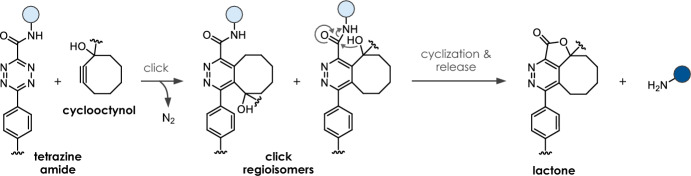
Cyclooctynol-triggered cleavage of tetrazine-amides following a click, cyclize, and release (CCR) process

Zheng et al. reported on the cleavage of tetrazine amides such as **C11** upon reaction with cyclooctynol triggers (e.g., **T16**-**T18**, Table 2). Following the IEDDA click reaction, release of the payload occurs spontaneously through intramolecular lactonization of the intermediate (Fig. 7) [36]. The click orientation with the hydroxy group at the side of the amide was found to be preferred, enabling higher release yields (> 80%). The introduction of substituents next to the hydroxy group results in lower ligation rates (substituted: *k*_*2*_ = 0.0075–0.065 M^−1^ s^−1^; not substituted: *k*_*2*_ = 0.25–0.36 M^−1^ s^−1^), but accelerates lactonization, and consequently, the release rate (substituted: *k*_*1*_ = 0.16–0.21 h^−1^; not substituted: *k*_*1*_ = 0.029 h^−1^), thus allowing for the fine-tuning of the overall process. Stability measurements revealed sufficient stability of the required tetrazine amides (76–81% intact after 24 h in PBS + 1 mM cysteine, 37 °C). Despite limited kinetics, this click-cyclize-release strategy (CCR) was demonstrated to achieve drug release inside mitochondria driven by organelle-specific accumulation [36].

**Table 2:**
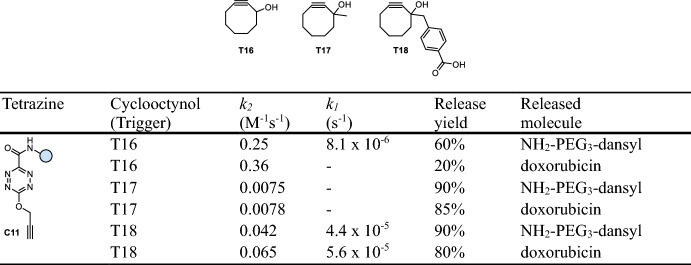
Chemical performance (*k*_*2*_, *k*_*1*_, release yield) of cyclooctynol-triggered cleavage of tetrazine amides

Reaction conditions: PBS/DMSO (9/1), room temperature [36]

### Isonitrile-Triggered Cleavage of Tetrazine Conjugates

**Fig. 8:**
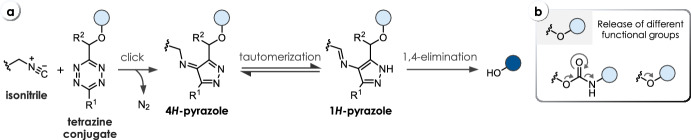
**a** Reaction mechanism of isonitrile-triggered cleavage of tetrazines. **b** Release of different functional groups, i.e., amines from Tz-carbamates and phenols from Tz-ethers

Franzini and co-workers utilized the unique chemistry of isonitriles to achieve cleavage of tetrazine-carbamates (**C12**, Table 3), enabling the release of amines and phenols (Fig. 8) [37]. While applying *n*-butyl isocyanide (*n-*BuNC) as trigger enables click-to-release in moderate rates and yields (*k*_*2*_ up to 0.3 M^−1^ s^−1^, *k*_*1*_ = 1.1 × 10^−5^ s^−1^, < 40%), trimethylsilylmethyl isocyanide (TMS-MeNC) achieves enhanced reaction kinetics and release yields (*k*_*2*_ up to ~ 0.8 M^−1^ s^−1^, *k*_*1*_ up to 3.5 × 10^−3^ s^−1^). Phenyl-substituted tetrazines (R^1^ = Ph) lead to a minor improvement of the overall release performance. Introducing a methyl or phenyl group onto the methylene linker (R^2^) accelerates click-to-release with TMS-MeNC but markedly reduces the release performance upon click reaction with *n*-BuNC. Release of phenols from Tz-ethers (**C13**) proceeds faster compared with the cleavage of Tz-carbamates for the release of amines (**C12**, Table 3). In general, all compounds exhibit good stability in human liver microsome as well as serum. Detailed nuclear magnetic resonance (NMR) studies and computational investigations revealed the mechanism of the cleavage reaction: initial isonitrile-Tz ligation leads to elimination of N_2_, affording a 4*H*-pyrazole, which tautomerizes to the 1*H*-pyrazole, leading to spontaneous elimination of the payload. However, the exact mechanism of the elimination step remains unclear. This bioorthogonal bond-cleavage reaction was tested in zebrafish embryos and demonstrated to enable dual release in combination with the cleavage of isonitriles (see chapter 5). Additionally, isonitrile-triggered cleavage of *t*BuTz-caged compounds was shown to be orthogonal to the TCO-triggered cleavage of *i*PrTz conjugates, enabling orthogonal bioorthogonal release inside cells [38].

**Table 3:**
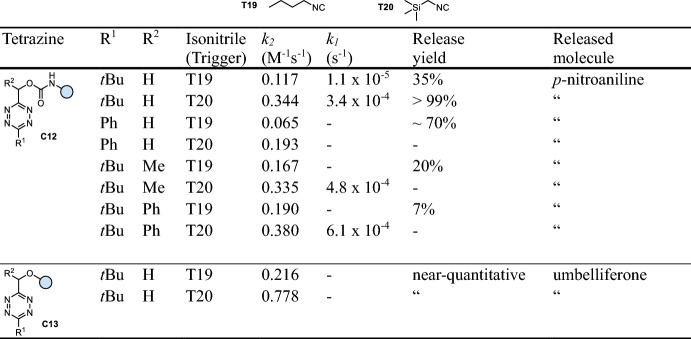
Chemical performance (*k*_*2*_, *k*_*1*_, release yield) of isonitrile-triggered cleavage of tetrazines

Reaction conditions: DMSO/PBS (4/1), 37 °C [37]

### *trans*-Cyclooctene-Triggered Cleavage of Tetrazine Conjugates

**Fig. 9:**
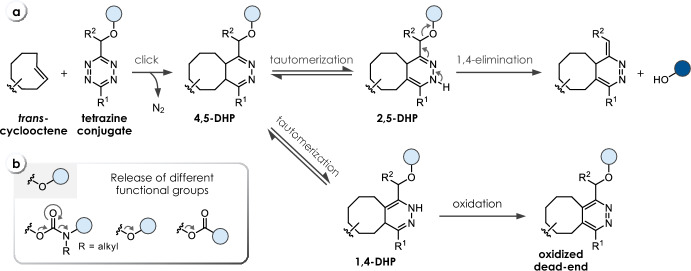
**a** Reaction mechanism of *trans*-cyclooctene (TCO)-triggered cleavage of tetrazines. The initial 4,5-DHP click product tautomerizes to a non-releasing 1,4-DHP and a releasing 2,5-DHP tautomer. **b** Release of different functional groups, i.e., secondary amines from Tz-carbamates, phenols from Tz-ethers, and carboxylic acids from Tz-esters

Efficient bioorthogonal cleavage of tetrazine conjugates triggered by the reaction with *trans*-cyclooctenes was first demonstrated by Robillard and co-workers (Fig. 9a) [39]. While unsubstituted carbamates were found to be insufficiently stable (half-life of degradation t_1/2_ < 6 min in MeCN/PBS at 37 °C), *N*-methyl carbamates (**C14**, Table 4) provided improved stability (t_1/2_ > 70 h in MeCN/PBS at 37 °C), enabling bioorthogonal release of secondary amines. In a detailed investigation of the underlying mechanism, the 2,5-dihydropyridazine tautomer (2,5-DHP) was identified as the releasing species. Furthermore, introduction of a methyl group at the methylene position (R^2^) was found to significantly enhance release. Compared with the previously developed Tz-triggered cleavage of TCO-conjugates, an acceleration of the click step upon reaction with unsubstituted TCO (Table 4) was observed, enabling near-complete release (93%). Further improvement of the click kinetics was achieved using a derivative of the highly reactive cyclopropane-fused *trans*-cyclooctene sTCO (Table 4), albeit resulting in a limited release yield (67%). The lower efficiency is hypothesized to result from slower tautomerization, leading to the formation of oxidative dead-end species. Application of this cleavage chemistry was successfully demonstrated in a biological environment using a click-cleavable antibody-Tz-drug conjugate [39].

The scope of TCO-triggered Tz-release was expanded by Wang et al., who demonstrated the release of phenols and carboxylic acids from Tz-ethers (**C13**) and Tz-esters (**C15**, Table 4), respectively (Fig. 9b) [40]. In general, equatorially configured TCO-5-OH achieves faster release rates and higher yields compared with axial TCO-5-OH, which might be attributed to the formation of different ratios of 2,5-DHP and 1,4-DHP tautomers. Methyltetrazine-ethers (R^1^ = Me) were shown to achieve complete release, whereas reactions of isopropyl- and phenyltetrazines result in lower release. In contrast, aliphatic Tz-esters achieve efficient release independent of the substitution (R^1^ and R^2^). In general, Me-substitution at the methylene linker (R^2^) was found to accelerate the release process. Aryl-Tz-esters achieve high release rates and higher yields, while showing good stability (> 85% after 24 h in MeCN/DMEM/FBS, 37 °C) [40].

**Table 4:**
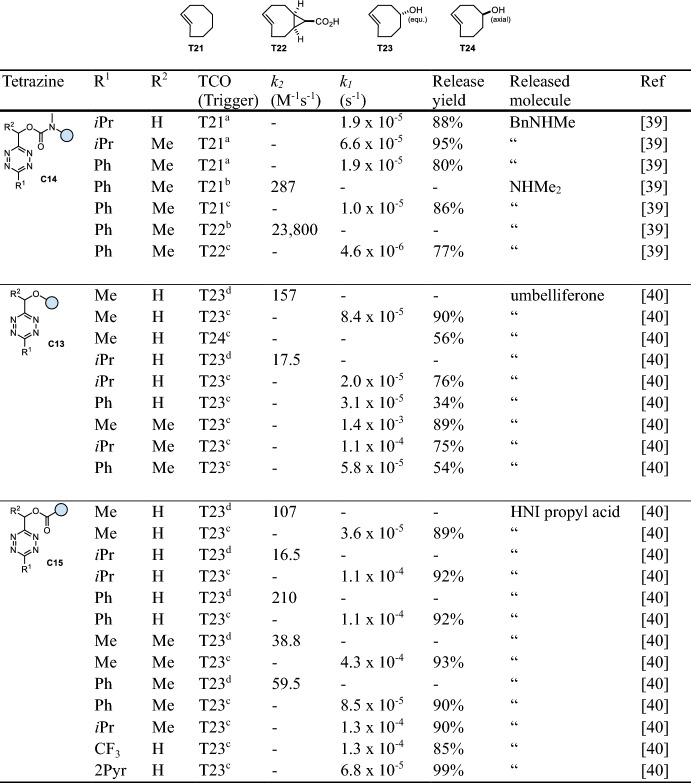
Chemical performance (*k*_*2*_, *k*_*1*_, release yield) of *trans*-cyclooctene-triggered cleavage of tetrazines

*HNI* 4-hydroxy-1,8-napthalimide

Reaction conditions: ^a^ PBS/MeCN (4/1), 37 °C; ^b^ PBS/MeCN (3/1), 20 °C; ^c^ PBS/MeCN (3/1), 37 °C; ^d^ PBS/MeCN (1/1), room temperature

## Tetrazine-Triggered Bioorthogonal Cleavage of Vinyl Ethers

**Fig. 10:**
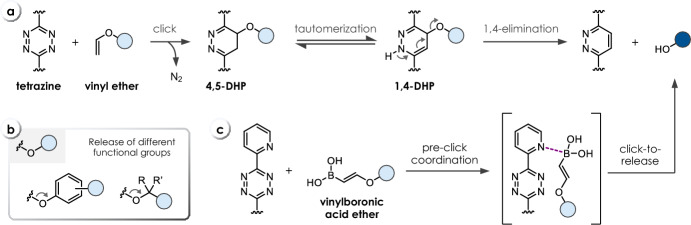
**a** Reaction mechanism of tetrazine-triggered cleavage of vinyl ethers. **b** Release of different functional groups, i.e., phenols and alcohols from vinyl ethers. **c** Improved click kinetics via pre-click coordination of vinylboronic acids ethers and 2-pyridyl-substituted tetrazines

On the basis of methods for the preparation of 1,2-diazines [41, 42], Devaraj and co-workers developed vinyl ether-caged phenols (**C16**, Table 5) as fluorogenic probes, which can be cleaved by reaction with 1,2,4,5-tetrazines to release fluorescent phenolic dyes (Fig. 10a) [43]. Tz-triggered cleavage of vinyl ethers has later been expanded to achieve release of alcohols (**C17**, Table 5, Fig. 10b) [44, 45], while self-immolative linker strategies were used to enable the release of amines from vinyl ether-caged phenols **C18** and **C19** (Table 5) [46, 47]. The IEDDA cycloaddition was revealed as the rate-limiting step, followed by a retro Diels–Alder reaction and fast elimination [45]. While the slow reaction kinetics of Tz-triggered vinyl ether cleavage limit its use in bioorthogonal bond-cleavage chemistry, it has been successfully exploited to develop proximity-enhanced and templated reactions [48, 49]. Importantly, the vinyl ether moiety has been reported to be sufficiently stable (up to 100% intact after 8 h in PBS at 37 °C) for applications in biological environments [45].

Bonger and co-workers achieved improved click kinetics through the development of vinylboronic acid ethers (**C20**, Table 5), which exhibited a fourfold increase in reactivity toward 2-pyridyl-substituted tetrazines, enabled by the pre-click coordination of both compounds (Fig. 10c) [50]. Recently, Chen and coworkers demonstrated that vinyl-caged tryptophan undergoes 1000-fold faster cleavage with **T1** compared with vinyl ether analogs [51].

**Table 5:**
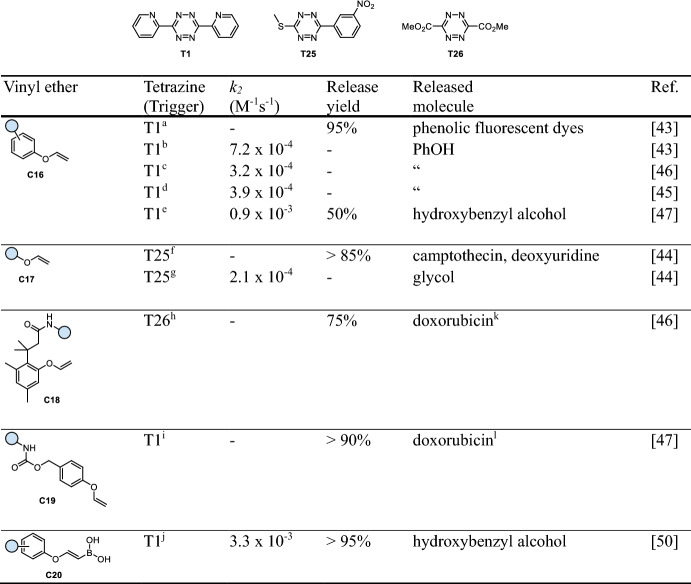
Chemical performance (*k*_*2*_, release yield) of tetrazine-triggered cleavage of vinyl ethers (release not rate-determining)

Reaction conditions: ^a^ MeOH/H_2_O (9/1), 65 °C; ^b^ DMSO/H_2_O (9/1), 37 °C; ^c^ DMSO/H_2_O (9/1), 25 °C; ^d^ DMF/H_2_O (9/1), 37 °C, ^e^ MeOH/PBS (3/1), 20 °C; ^f^ MeCN/MeOH/H_2_O (4/5/1), 37 °C; ^g^ DMSO/H_2_O (1/1), 37 °C; ^h^ DMSO, 37 °C; ^i^ PBS/MeCN, 37 °C, ^j^ MeOH/PBS (3/1), room temperature. ^k^ Resulting from self-immolation (intramolecular lactonization) of the linker upon vinyl ether cleavage. ^l^ Resulting from self-immolation (1,6-elimination) of the linker upon vinyl ether cleavage

## Tetrazine-Triggered Bioorthogonal Cleavage of 3-Isocyanopropyl Derivatives

**Fig. 11:**
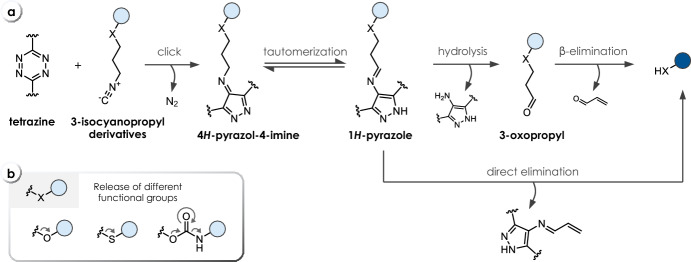
**a** Reaction mechanism of tetrazine-triggered cleavage of 3-isocyanopropyl derivatives. After initial ligation, the formed 4*H*-pyrazol-4-imine tautomerizes to an aromatic 1*H*-pyrazole. The payload is released either after hydrolysis from 3-oxopropyl derivatives or directly from the 1*H*-pyrazole through β-elimination. **b** Release of different functional groups, i.e., phenols and thiophenols from ethers and thioethers, respectively, and amines/anilines from carbamates

Leeper et al. were first to further analyze the previously reported [4 + 1] cycloaddition of isonitriles (isocyanides) with tetrazines [52] toward its application for biocompatible ligation [53]. Even though their study focused on ligation rather than release chemistry, the initial results shed light onto the necessary chemical composition of such systems for efficient click-to-release applications. They identified tertiary isonitriles as ideal candidates for ligation, as subsequent tautomerization and thus elimination reactions were rendered impossible (vide infra). In contrast, primary isonitriles, such as *n*-butyl isocyanide (**C21**), and secondary isonitriles were shown to expel aldehydes upon cycloaddition with 1,2,4,5-tetrazines in aqueous media. As an exception, methyl isocyanopropionate, a primary isonitrile, was shown to release very slowly due to tautomerization to the stable vinylogous urethane. To establish a more versatile chemistry for the release of cargo molecules, Franzini et al. employed 3-isocyanopropyl derivatives (Fig. 11a) [54]. These compounds react with tetrazines to initially form non-isolatable tetraazanorbornadienimines, which spontaneously undergo a [4 + 2]-cycloreversion with release of N_2_, resulting in 4*H*-pyrazol-4-imine derivatives. Tautomerization leads to 1*H*-pyrazole intermediates, which in the presence of water hydrolyze to 3-oxopropyl derivatives and 4-aminopyrazoles. Spontaneous cleavage of the 3-oxopropyl moieties via β-elimination leads to elimination of the attached payloads such as phenols (from ethers **C22** or **C23**, Table 6) and thiophenols (from thioethers **C26**) as well as anilines and amines (from carbamates **C24** or **C25**) under physiological conditions (Fig. 11b). Mechanistic investigations provided compelling evidence that hydrolysis of the imine might not be necessary, as β-elimination was proven to occur from 1*H*-pyrazol-4-imines when reacted with sterically demanding 3,6-bis(*tert*-butyl)-1,2,4,5-tetrazine (*t*Bu_2_Tz). Hence, both release pathways (β-elimination of the 3-oxopropyl moiety and direct elimination of the 1*H*-pyrazole) might be operating [55]. Kinetic investigations revealed that the click reaction of the PEGylated bis(2-pyridyl)-substituted tetrazine **T1a** is significantly faster with ether-linked isonitriles **C22** than with carbamate derivatives **C24** (*k*_*2*_ = 4.0 M^−1^ s^−1^ versus 1.1 M^−1^ s^−1^, 10% DMSO in PBS, Table 6). Tautomerization and hydrolysis of the imine were found to proceed very rapidly under aqueous conditions and did not contribute to the overall rate of the reaction. A similar trend as the initial click was observed for the rate-determining β-elimination of the intermediate aldehydes, with the release of phenols being approximately threefold faster than the release of amines (1.6 × 10^−4^ s^−1^ for **C22** versus 5 × 10^−5^ s^−1^ for **C24**, Table 6). When performing the reaction in PBS/serum (1/1), a significant acceleration of the elimination process was observed, which was attributed to the beneficial catalysis by serum albumins [56]. Click-triggered bond-cleavage leading to the release of different cargos (phenols from **C22**/**C23**, thiophenols from **C26**, amines from carbamates **C24**/**C25**) was tested in PBS/DMSO (1/1), revealing high release yields (71–96%) after 3 h.

Modification of the isopropyl chain with anion-stabilizing phenyl groups at the 2-position was found to enable a substantial acceleration of the elimination, making the click reaction the rate-determining step [55]. For instance, a ~ tenfold higher release rate constant (*k*_*1*_) was observed upon reacting **T1** with phenyl-substituted **C23** and **C25**, compared with isonitriles **C22** and **C24**, respectively (Table 6).

However, the slow click kinetics as well as the unfavorable stability of bis-pyridyl-substituted tetrazines such as **T1** and **T1a** motivated the optimization of the Tz scaffold, eventually leading to the discovery of an atypical structure–activity relationship for the reaction of tetrazines and isonitriles. Sterically more hindered *t*Bu-substituted tetrazines were found to show increased reactivity, enabling a four- to eightfold faster reaction when using 3-*tert*-butyl-6-pyrimidine-tetrazines **T27a-c** (Table 6) [57]. The second-order rate constant of the reaction with PhEtNC (**C27**) in DMSO/PBS (4/1) could thereby be increased from 0.3 M^−1^ s^−1^ (**T1**) to 1.15 M^−1^ s^−1^ (**T27a**), 1.53 M^−1^ s^−1^ (**T27b**), and 2.42 M^−1^ s^−1^ (**T27c**). A higher water content further boosted the reaction with Tz **T27c** up to a rate constant of 29.4 M^−1^ s^−1^ (Table 6). These tetrazines were moreover found to achieve efficient release (e.g., 99% after reacting **T27c** with **C22**, Table 6) and exhibit a relatively high stability in buffered solution containing glutathione (71% intact **T1** compared with 99% intact **T27a** after 3 h).

**Table 6:**
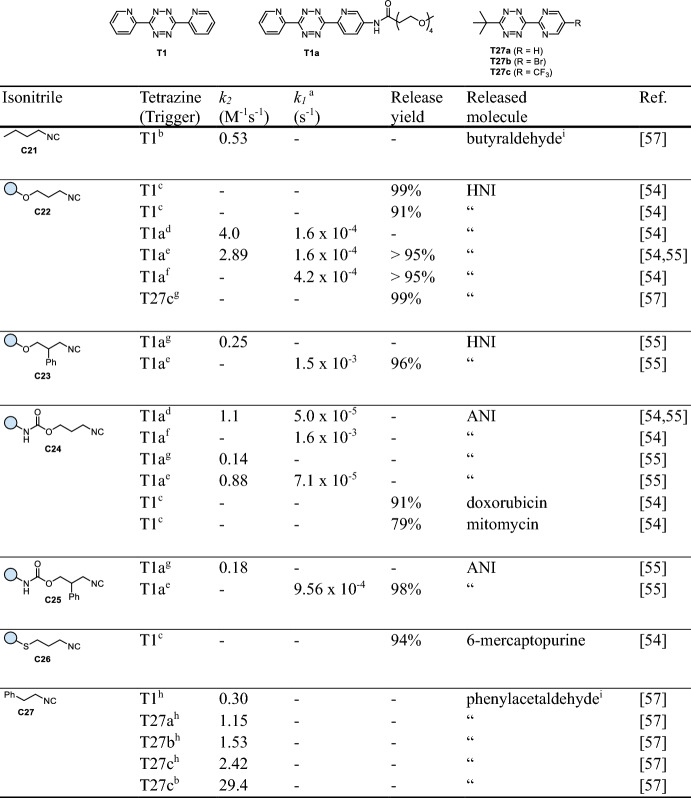
Chemical performance (*k*_*2*_, *k*_*1*_, release yield) of tetrazine-triggered cleavage of isonitriles

*HNI* 4-hydroxy-1,8-napthalimide; *ANI* 4-amino-1,8-napthalimide

^a^ Rate constant for the entire release process (incl. further elimination of the 1*H*-pyrazole intermediate; *cf.* Figure 11). Reaction conditions: ^b^ H_2_O/DMSO (4/1), 37 °C; ^c^ PBS/DMSO (1/1), 37 °C; ^d^ PBS/DMSO (9/1), 37 °C; ^e^ PBS/DMSO (4/1), 37 °C; ^f^ PBS/serum (1/1), 37 °C; ^g^ PBS/DMSO (1/4), 37 °C; ^h^ H_2_O/DMSO (1/4), 37 °C; ^i^ Carbonyl compounds that do not undergo β-elimination

## Click-Triggered Bioorthogonal Cleavage of Iminosydnones

**Fig. 12:**
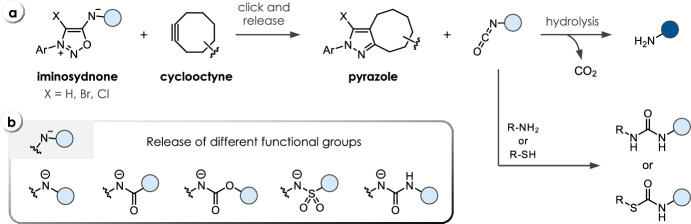
**a** Cyclooctyne-triggered cleavage of iminosydnones. The released isocyanate moiety can either hydrolyze to yield NH_2_ functionalities (e.g., amines, amides, sulfonamides) or be intercepted by endogenous amines or thiols, leading to the formation of urea or thiocarbamate products, respectively. **b** Release of different functional groups

Mesoionics—five-membered heterocycles with two opposite charges [58]—were identified as versatile tools for bioconjugation reactions, owing to their exceptional reactivity with strained cycloalkynes in strain-promoted iminosydnone-cyclooctyne cycloadditions (SPICC). Bernard et al. were first to conduct a comprehensive analysis of various mesoionic compounds, establishing a reactivity profile with the most commonly used strained cycloalkynes [59]. They identified dithiolium compounds, sydnones, and iminosydnones as the most optimal reaction partners. Notably, iminosydnones emerged as particularly suitable for the click-to-release field as these compounds liberate isocyanates instead of CO_2_ or COS. Mechanistically, the initial click reaction with the cyclic alkyne forms bicyclic intermediates, which rapidly evolve into the corresponding pyrazoles via the elimination of isocyanates through a retro Diels–Alder reaction. Subsequent fast hydrolysis and decarboxylation lead to release of the final products (Fig. 12). Electronically stabilizing cargo moieties (e.g., aryl groups), however, can render the isocyanate less susceptible to hydrolysis. These isocyanates react with biogenic thiols or amines to form thiocarbamate or urea derivatives in biological media (Fig. 12). The click kinetics were found to be heavily influenced by the cargo moiety attached to the exocyclic nitrogen. The reactions of iminosydnone amides (**C32**) with the cyclooctyne BCN (bicyclo[6.1.0]non-4-yne, **T28**) showed the lowest second-order rate constants, followed by carbamates (**C30**), sulfonamides (**C31**), and urea derivatives (**C28**, **C29**) (Table 7). Introduction of electron-withdrawing substituents (such as *p-*ethoxycarbonylphenyl) increased the reaction rate of iminosydnone (e.g., 0.18 M^−1^ s^−1^ versus 0.008 M^−1^ s^−1^ with a *p-*hydroxyphenyl substituent in the reaction of **C28** and **T28**; Table 7). Further acceleration was achieved by using the more reactive cycloalkynes DBCO (dibenzocyclooctyne, **T29**) and TMTH (tetramethylthiacycloheptyne, **T30**), reaching rate constants of 1.47 M^−1^ s^−1^ and 29.2 M^−1^ s^−1^, respectively, however, also resulting in a reactivity/stability tradeoff [60, 61]. Similar to other cycloaddition reactions, an increased water content was found to be beneficial, for instance, doubling the rate when switching from 80% DMSO to 20% DMSO in PBS (e.g., 1.47 versus 3.15 M^−1^ s^−1^ for **C28** with DBCO, Table 7). Bromine substitution in **C29** increased the reactivity approximately twofold with DBCO and more than 16-fold with BCN [62, 63]. Chlorination was later found to be even more beneficial, increasing *k*_*2*_ up to threefold compared with the Br-substituted analogs, finally enabling click-to-release of sulfonamides (**C31**) and amides (**C32**) [64]. Computational studies carried out by Houk et al. revealed a lowered distortion energy as the origin of this halogen effect [65].

The fastest SPICC reactions have been reported for iminosydnones with aryl and alkyl substituents attached to the exocyclic nitrogen (**C33** and **C34**) [66]. These derivatives exhibit a rate constant of up to 1200 M^−1^ s^−1^ for benzyl-substituted **C34** with DBCO, albeit at an increased pH of 9.5. The speed of the reaction was found to be heavily correlated with the pK_a_ of the nitrogen atom. Evolution of negative charge (and thus necessary deprotonation) at this position (pK_a_ = 8.7 for *N*-benzyl) was deemed to be crucial for fast cycloaddition. Reactions at physiological pH of 7.4 lowered the rate constant for the reaction of **C34** and DBCO, and down to 2.5 M^−1^ s^−1^ at pH 5. The phenyl-substituted scaffold **C33** showed a lower pH dependency, and a surprising reactivity increase in crude cell lysate up to 1395 M^−1^ s^−1^, compared with 240 M^−1^ s^−1^ in PBS. However, due to the strong electron-donating effect of both cargos, only slow hydrolysis to the corresponding primary amines and anilines was observed. The system thus allowed the release of isocyanate electrophiles inside living cells, which were rapidly intercepted by endogenous glutathione or lysine residues. A new SPICC approach was introduced by Lui et al., who circumvented the known instability of silacycloheptynes by masking the alkyne as a photocleavable cyclopropenone (**T31**, Table 7) [67]. Upon irradiation, the resulting cycloalkyne rapidly reacts with iminosydnones (*k*_*2*_ up to 22.2 M^−1^ s^−1^), for instance, enabling 85% prodrug activation of celecoxib in seconds at 20 µM concentrations.

**Table 7:**
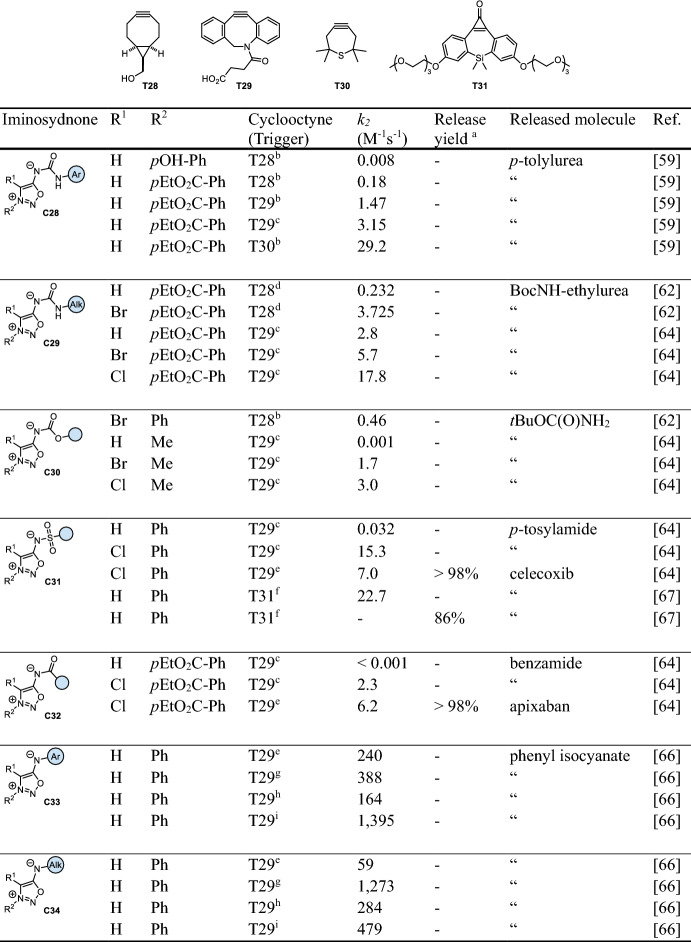
Chemical performance (*k*_*2*_, release yield) of cyclooctyne-triggered cleavage of iminosydnones

^a^ The cited references do not provide specific release yields but focus on click kinetics. In general, release from iminosydnones is near-complete in most cases. [59] Reaction conditions: ^b^ PBS/DMSO (1/4), 25 °C; ^c^ PBS/DMSO (4/1), 25 °C; ^d^ PBS, 25 °C; ^e^ PBS/DMSO (4/1), 37 °C; ^f^ MeCN, 25 °C; ^g^ pH 9.5 buffer/DMSO (4/1), 37 °C; ^h^ human plasma/DMSO (4/1), 37 °C; ^i^ cell lysate/DMSO (4/1), 37 °C. Unless otherwise noted, the DMSO content is < 2%

## Click-Triggered Bioorthogonal Cleavage of Aryl Azides

**Fig. 13:**
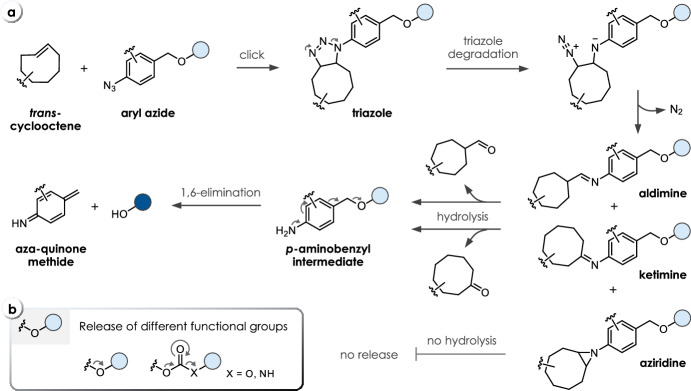
**a** Reaction mechanism of *trans*-cyclooctene-triggered cleavage of aryl azides. **b** Release of different functional groups, i.e., alcohols and phenols from ethers and carbonates as well as amines from carbamates

The 1,3-dipolar cycloaddition reaction between azides and strained *trans*-cyclooctenes (TCOs) has been mostly overlooked in bioorthogonal chemistry due to the instability of its products [68]. Gamble et al. took advantage of this undesired property to achieve click-to-release of prodrugs through incorporation of a self-immolative *p*-aminobenzyloxycarbonyl (PABC) linker (Fig. 13a) [68, 69]. The reaction of the aryl azide with a TCO leads to the formation of an initial triazole click product. Depending on the substitution of the aryl azide, the triazole intermediate undergoes ring contraction with the concomitant liberation of N_2_ to form aldimines as predominant products. Ketimines as well as hydrolytically stable aziridines are also formed under these conditions. Acid-catalyzed hydrolysis of both imines leads to formation of the respective carbonyl products and *p*-aminobenzyl intermediates (ethers, carbonates, or carbamates), which finally release the payload (Fig. 13b) through 1,6-elimination, yielding aza-quinone methides as byproducts [70]. Initial optimization studies focused on the modification of the aromatic core structure due to the low second-order rate constants (0.02 M^−1^ s^−1^) observed for unmodified 4-azidobenzyl carbamates **C35** and TCOs, such as equatorial (**T23**) and axial TCO-5-OH (**T24**). The addition of fluorine atoms to 4-azidebenzyl carbonates (**C36**) and ethers (**C37**) led to a sixfold acceleration of the reaction with **T23** (Table 8). However, the opposite trend was observed for the subsequent release step [71]. Electron-withdrawing substituents caused a drop in the pK_a_ value (from 8 to 5) of the intermediate imine, slowing acid-catalyzed hydrolysis under physiological conditions, and decelerated the self-immolation due to the destabilization of the benzylic carbon. In-depth NMR studies revealed that the 1,6-elimination became rate-determining for the perfluorinated analogues. To tackle the slow release, a second generation of aryl azides, including pyridines (**C38**) and compounds with a stabilizing methyl group at the benzylic position of **C37** (Table 8), were tested with **T23**, **T24** and the more strained *trans*-cyclooctene dTCO (***anti***/***syn*-T32**) [72]. While pyridyl azides proved to be unsuitable for bioorthogonal reactions with significantly decreased elimination rates (t_1/2_ = 3.5 days for **C38**), methylation improved the kinetics of the release step (t_1/2_ = 42 min instead of 4.8 h) without impacting the click kinetics (Table 8). Exchanging the trigger from **T23** to ***syn*-T32** and increasing the water content (from 50% to ~ 95% PBS, in mixtures with MeCN) led to an almost 300-fold acceleration of the reaction with perfluorinated azidobenzyl ethers (**C37**) from a second-order rate of 0.02 M^−1^ s^−1^ (**T23**) to 4.9 M^−1^ s^−1^ (***syn*-T32**). This change, paradoxically, led to slower elimination, resulting in a 1.3- to 1.7-fold increase in the time required to achieve maximum release. This observation was proposed to be the result of increased triazoline and/or aldimine stability. Nevertheless, considering the rate constant of 4.9 M^−1^ s^−1^, this reaction represents the fastest cycloaddition of an azide and a strained TCO reported thus far [72].

TCO-triggered cleavage of aryl azides was applied to achieve (i) indirect release of drugs by destabilizing a diphenylalanine hydrogel [73, 74], (ii) bioorthogonal decaging of a masked lysine residue to turn on the activity of wild-type firefly luciferase [75], and (iii) pretargeted prodrug activation using antibodies modified with **T23** or ***syn*-T32** and aryl azide-caged doxorubicin (Table 8) [76]. While release studies revealed quantitative reaction of the prodrug with dTCO-modified antibody, only limited doxorubicin release of approximately 20% was detected. The hydrophobic environment of the antibody surrounding the more apolar post-click products was hypothesized to impede the hydrolysis and final release of the drug [76].

**Table 8:**
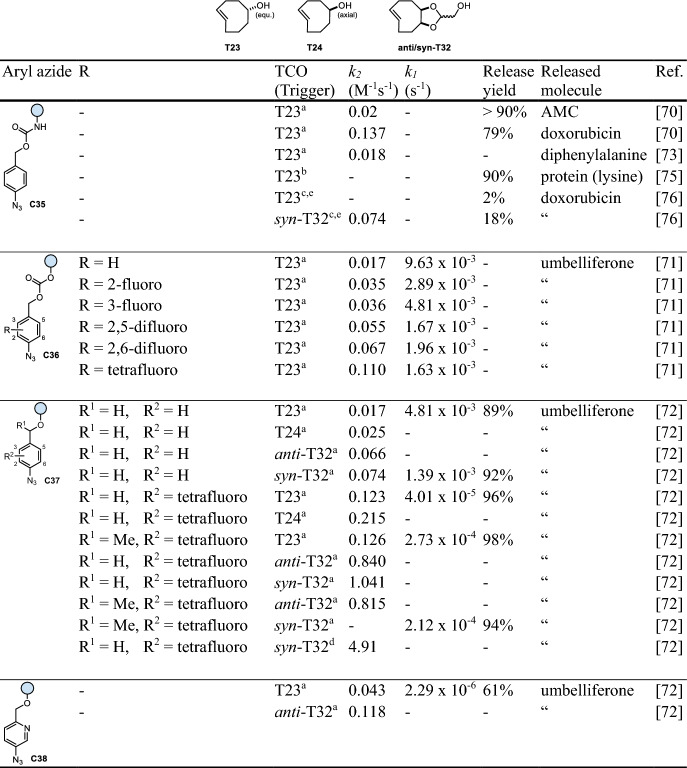
Chemical performance (*k*_*2*_, *k*_*1*_, release yield) of *trans*-cyclooctene-triggered cleavage of aryl azides

*AMC* 7-amino-4-methylcoumarin

Reaction conditions: ^a^ PBS/MeCN (1/1), 37 °C; ^b^ cell lysate; ^c^ PBS; ^d^ PBS/MeCN (18/1), 37 °C. ^e^ TCO attached to antibody. Unless otherwise noted, the DMSO content is < 2%

## Click-Triggered Bioorthogonal Cleavage of Benzonorbornadienes

**Fig. 14:**
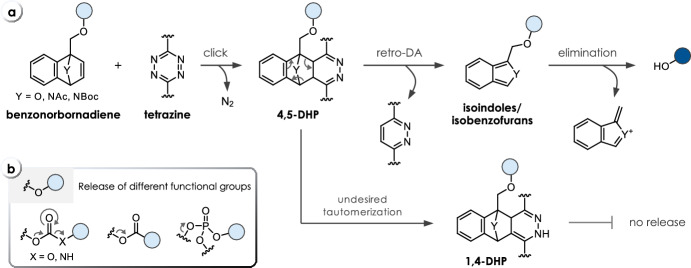
**a** Reaction mechanism of tetrazine-triggered cleavage of benzonorbornadienes. After IEDDA ligation (click), the resulting 4,5-dihydropyridazine (4,5-DHP) undergoes a retro-Diels–Alder cycloreversion leading to isoindoles/isobenzofurans, and subsequently, to release of the payload. **b** Release of different functional groups, i.e., alcohols and amines from carbonates and carbamates, respectively, as well as carboxylic acids and phosphoric acid derivatives

IEDDA reactions of *O*- or *N*-bridged benzonorbornadienes with tetrazines form 4,5-dihydropyridazine intermediates. After the rate-limiting cycloaddition, the liberation of 1,2-pyridazine via a retro Diels–Alder cycloreversion leads to formation of highly reactive isobenzofurans or isoindoles, respectively (Fig. 14a) [77]. The isobenzofuran/isoindole intermediates can further undergo elimination to release a variety of payloads attached to the benzonorbornadiene via a methylene linker (Fig. 14a). Release of amines from carbamates (**C39**), alcohols from carbonates (**C41**), carboxylic acids (**C42**), and phosphoric acid derivatives (**C43**) from the respective esters has been reported (Fig. 14b, Table 9) [78]. The choice of the bridgehead atom was shown to be very crucial. *C*-bridged norbornadienes form 1,4-DHP tautomers, which do not lead to any further elimination (Table 9). This side reaction was also described for certain *O*-bridged norbornadienes upon reaction with tetrazine **T1** [79]. Formation of the 1,4-DHP tautomer could be prevented at higher reaction temperatures, as the transition states of the retro Diels–Alder reactions were shown to have an elevated entropy of activation. Interestingly, this effect was not observed with *N*-bridged systems, which was attributed to the higher dienofugacity of isoindoles relative to isobenzofurans [80]. Therefore, generally higher release yields have been reported for *N*-bridged systems. Unfortunately, *N*-bridged benzonorbornadienes are about 2.5-fold less reactive compared with their *O*-bridged analogs. Unsubstituted *O*-bridged benzonorbornadienes without any payload exhibited a 12-fold higher IEDDA reactivity. Introduction of a methyl substituent at the methylene position (**C40**) resulted in a lower second-order rate constant. For instance, methylation led to a reduction of the rate constant from 0.058 M^−1^ s^−1^ to 0.017 M^−1^ s^−1^ for the reactions of the respective *O*-bridged compounds (**C39**, **C40**) with tetrazine **T33** (Table 9) [78]. Increased steric bulk at the bridgehead position by using *N*-Boc-protected derivatives also halved the reaction rate (0.0084 M^−1^ s^−1^ versus 0.017 M^−1^ s^−1^ for the *N*-Ac analog, Table 9) [77]. Therefore, *O*- and *N-*Ac-bridged benzonorbornadienes have been used for bioorthogonal click-to-release reactions, for instance, to achieve activation of a doxorubicin prodrug [77] and the release of siRNA [81].

**Table 9:**
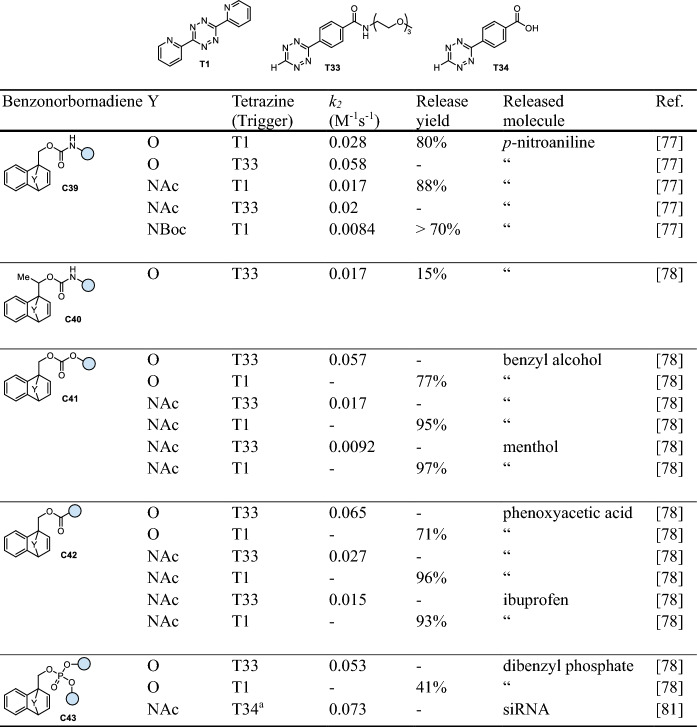
Chemical performance (*k*_*2*_, release yield) of tetrazine-triggered cleavage of benzonorbornadienes

Reaction conditions unless otherwise noted: PBS/DMSO (1/9), 37 °C. ^a^ PBS/DMSO (9/1), 37 °C

## Click-Triggered Bioorthogonal Cleavage of Cyclopentadienones

**Fig. 15:**
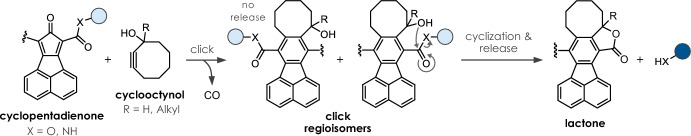
**a** Cyclooctynol-triggered cleavage of cyclopentadienones. The initial IEDDA ligation leads to the formation of two regioisomers as click products, with only one being able to release the payload through intramolecular cyclization

Wang et al. developed a bioorthogonal release system on the basis of cyclopentadienone-alkyne chemistry by combining a cascade of three reactions: IEDDA cycloaddition, followed by cheletropic extrusion and subsequent lactonization facilitate a simultaneous release of CO and the payload (Fig. 15). The initially developed intramolecular system showed a half-life of release of 0.66 h at pH 7.0 [82]. The methodology was then extended to an intermolecular click reaction using cyclooct-2-yn-1-ol as trigger, achieving a second-order rate constant of 0.018 M^−1^ s^−1^ [83]. Improvement of the click reaction rate was achieved by replacing the phenyl substituents on the cyclopentadienone with more electron-withdrawing methyl ester or morpholine-4-carbonyl groups, increasing the second-order rate constant up to 0.17 M^−1^ s^−1^. Due to the uncontrolled regiochemistry of the click reaction, release is limited to about 50% [83, 84]. Applying the more strained cyclooctyne BCN further improved *k*_*2*_ (up to 12.2 M^−1^ s^−1^), which was, however, only used for ligation purposes [84].

## Conclusions

Alongside the discovery of fast and selective bioorthogonal ligation reactions, the concept of bioorthogonal bond-cleavage has gained significant attention, primarily for its potential to achieve spatiotemporal control of (bio)molecular function. Although the kinetics of the initial click reaction are often the main focus, subsequent processes leading to release are equally critical for overall speed and yield of the reaction. Importantly, a fast click does not guarantee rapid or complete release. Mechanistic insights into post-click processes, such as tautomerization, elimination, or cyclization, are essential for optimizing reaction conditions and designing bioorthogonal reaction partners. Moreover, the stabilities of initial click products and intermediates must be considered to ensure complete reactions within desired timeframes. Accounting for these factors enhances the utility of bioorthogonal click-to-release strategies, paving the way for next-level applications in chemical biology, medicinal chemistry, and beyond.

## Data Availability

No datasets were generated during the current study. The data summarized and discussed in this review were taken from the cited references and converted to other units if required.
